# Vitamin K2 Modulates Vitamin D‐Induced Mechanical Properties of Human 3D Bone Spheroids In Vitro

**DOI:** 10.1002/jbm4.10394

**Published:** 2020-08-03

**Authors:** Maria Schröder, Elisabeth Aurstad Riksen, Jianying He, Bjørn Helge Skallerud, Mona Elisabeth Møller, Aina‐Mari Lian, Unni Syversen, Janne Elin Reseland

**Affiliations:** ^1^ Department of Biomaterials University of Oslo Oslo Norway; ^2^ Department of Structural Engineering, Faculty of Engineering Norwegian University of Science and Technology (NTNU) Trondheim Norway; ^3^ Axial Vita AS Oslo Norway; ^4^ Oral Research Laboratory, Institute for Clinical Dentistry University of Oslo Oslo Norway; ^5^ Department of Clinical and Molecular Medicine NTNU Trondheim Norway; ^6^ Department of Endocrinology, Clinic of Medicine, St. Olavs Hospital Trondheim University Hospital Trondheim Norway

**Keywords:** BONE STIFFNESS, OSTEOBLASTS, OSTEOSPHERES, VITAMIN D, VITAMIN K2

## Abstract

Rotational culture promotes primary human osteoblasts (hOBs) to form three‐dimensional (3D) multicellular spheroids with bone tissue‐like structure without any scaffolding material. Cell‐based bone models enable us to investigate the effect of different agents on the mechanical strength of bone. Given that low dietary intake of both vitamin D and K is negatively associated with fracture risk, we aimed to assess the effect of these vitamins in this system. Osteospheres of hOBs were generated with menaquinone‐4 (MK‐4; 10μM) and 25‐hydroxyvitamin D_3_ [25(OH)D_3_; 0.01μM], alone and in combination, or without vitamins. The mechanical properties were tested by nanoindentation using a flat‐punch compression method, and the mineralized extracellular bone matrix was characterized by microscopy. The in vitro response of hOBs to MK‐4 and 25(OH)D_3_ was further evaluated in two‐dimensional (2D) cultures and in the 3D bone constructs applying gene expression analysis and multiplex immunoassays. Mechanical testing revealed that 25(OH)D_3_ induced a stiffer and MK‐4 a softer or more flexible osteosphere compared with control. Combined vitamin conditions induced the same flexibility as MK‐4 alone. Enhanced levels of periostin (*p* < 0.001) and altered distribution of collagen type I (COL‐1) were found in osteospheres supplemented with MK‐4. In contrast, 25(OH)D_3_ reduced COL‐1, both at the mRNA and protein levels, increased alkaline phosphatase, and stimulated mineral deposition in the osteospheres. With the two vitamins in combination, enhanced gene expression of periostin and COL‐1 was seen, as well as extended osteoid formation into the central region and increased mineral deposition all over the area. Moreover, we observed enhanced levels of osteocalcin in 2D and osteopontin in 3D cultures exposed to 25(OH)D_3_ alone and combined with MK‐4. In conclusion, the two vitamins seem to affect bone mechanical properties differently: vitamin D enhancing stiffness and K2 conveying flexibility to bone. These effects may translate to increased fracture resistance in vivo. © 2020 The Authors. *JBMR Plus* published by Wiley Periodicals LLC on behalf of American Society for Bone and Mineral Research.

## Introduction

Three‐dimendional (3D) bone spheroids, also referred to as osteospheres, represent new in vitro models to study the molecular mechanisms of bone remodeling,^(1^ ^)^ as well as the pathophysiology of bone diseases and healing.^(^ ^2^ ^)^ Slow horizontal clinorotation promotes aggregation and differentiation of bone cells into bone tissue‐like structures without the inclusion of any scaffold material.^(^ ^1^, ^3^, ^4^, ^5^ ^)^ Under these culture conditions, primary human osteoblasts (hOBs) form a self‐assembled mineralized extracellular matrix within the 3D bone spheroids.^(^ ^1^ ^)^ We have previously shown that these spheroids represent a suitable model for assessment of the effect of various stimuli on the biomechanical properties of bone.^(^ ^6^ ^)^

Vitamin D stimulates the absorption of calcium and phosphate from the intestine. Low serum vitamin D levels induce secondary hyperparathyroidism, leading to increased bone resorption, decreased BMD, and a higher fracture incidence.^(^ ^7^ ^)^ Vitamin D is mainly synthesized in the skin after exposure to sunlight, but is also obtained from dietary sources. Vitamin D is metabolized to 25‐hydroxyvitamin D_3_ [25(OH)D_3_] in the liver, and to 1,25‐dihydroxyvitamin D_3_ [1,25(OH)_2_D_3_] in the kidneys by the enzyme 1α‐hydroxylase.^(^ ^8^ ^)^ 1,25(OH)_2_D_3_ is the biologically active form,^(^ ^9^ ^)^ whereas 25(OH)D_3_ levels are used as a measure of vitamin D status.^(^ ^10^ ^)^ 1α‐hydroxylase, as well as the vitamin D receptor, is also expressed in osteoblasts.^(^ ^11^, ^12^, ^13^, ^14^, ^15^ ^)^ For studies of the effect of vitamin D on osteoblasts in vitro, 25(OH)D_3_ is preferred over 1,25(OH)_2_D_3_ because of its longer half‐life time.^(^ ^16^ ^)^

Menaquinones, referred to as vitamin K2, are a family of molecules consisting of a 2‐methyl‐1,4‐naphthoquinone structure with a variable number of 3′‐substituted isoprene units.^(^ ^17^ ^)^ The main dietary menaquinones are MK‐4 to MK‐10, which are found in fermented food and animal products.^(^ ^18^, ^19^ ^)^ Vitamin K‐dependent proteins have been isolated in bone, cartilage, kidney, and vascular and soft tissues.^(^ ^20^ ^)^ These proteins include, among others, osteocalcin (OC) and periostin.^(^ ^21^ ^)^ OC gene expression is regulated by 1,25(OH)_2_D_3,_^(^ ^22^ ^)^ whereas the protein's capability to bind to calcium relies on the vitamin K‐dependent gamma‐carboxylation of three glutamic acid residues in the molecule.^(^ ^23^ ^)^ Periostin is a matricellular protein involved in the regulation of collagen fibril diameter and cross‐linking.^(^ ^24^ ^)^ Vitamin K2 also exerts direct effects on bone cells, stimulating osteoblastogenesis^(^ ^25^, ^26^, ^27^ ^)^ and inhibiting the osteoclast differentiation.^(^ ^25^, ^27^ ^)^ Vitamin K2 has been reported to bind to the steroid and xenobiotic receptor (SXR), resulting in enhanced expression of several components of the bone matrix.^(^ ^26^ ^)^ Low vitamin K intake, as well as high levels of undercarboxylated OC (unOC), is associated with an increased risk of bone fragility concomitant with hip fractures in elderly patients.^(^ ^28^, ^29^, ^30^ ^)^

The vitamin K2 synthetic form MK‐4 is approved in antiosteoporosis therapy in Japan and is frequently used in combination with bisphosphonates.^(^ ^31^ ^)^ However, the effect of MK‐4 on BMD and fracture risk remains a controversy.^(^ ^32^ ^)^ Combined administration of vitamin D and K is suggested to have synergistic positive effects on calcium homeostasis and bone and cardiovascular health.^(^ ^33^ ^)^ Vitamin D enhances vitamin K‐dependent bone protein production.^(^ ^34^, ^35^ ^)^ Both vitamin D and K have been demonstrated to be cofactors in the gamma‐carboxylation of OC.^(^ ^36^, ^37^ ^)^ An increasing number of randomized controlled trials have also evaluated the combined treatment of vitamin K2 and D with different outcomes.^(^ ^38^, ^39^, ^40^ ^)^

Both vitamins D and K play important roles in bone health; however, their combined effects on mechanical properties of 3D bone spheroids have, to our knowledge, not been studied before. Therefore, we wanted to investigate the in vitro effects of vitamin D and K, alone and in combination on the biomechanical properties of 3D bone spheroids of primary hOBs. To elucidate the molecular mechanisms, we aimed at identifying the effect of these vitamins on the gene expression and secretion of proteins and cytokines involved in the biological and mechanical functions of bone in both 2D cell cultures of primary hOBs and in 3D bone constructs.

## Materials and Methods

### 2D Cell cultures

Commercially available primary hOBs (NHOst cell system; Lonza, Walkersville, MD, USA) were grown in osteoblast growth medium (OGM; Lonza) at 37°C in a humidified atmosphere of 95% air and 5% CO_2_. The medium was changed three times weekly, and the cells were subcultured and seeded in 24‐well‐plates. At confluence, synthetic vitamin K2, MK‐4 (at 1μM and 10μM; gift from Kappa Biosciences, Oslo, Norway), and 25(OH)D_3_ (0.01μM; Calcifediol CRS; European Pharacopoeia Reference Standard, EDQM, Strasbourg, France) were added alone or in combination to the culture medium. Cells cultured with regular OGM were used as control. Cell culture media were harvested after 1, 7, 14, and 20 days of incubation.

### Generation of 3D osteospheres

Primary hOBs (Lonza) were cultured in OGM (PromoCell, Heidelberg, Germany) with supplement mix (PromoCell) and 100 U mL^−1^ penicillin and 100 μg/mL^−1^ streptomycin (PAA Laboratories GmbH, Pasching, Austria). hOBs (>3 × 10^6^ cells) were inoculated into CelVivo 10‐mL bioreactors (Cat. no. DM 010; CelVivo, Blommenslyst, Denmark), and osteospheres were generated in the BioArray Matrix drive BAM v4 (CelVivo) in a humidified atmosphere with 5% CO_2_ at 37 °C at a rotation speed of 4 rpm. On culture day 7, the medium was supplemented with 10mM β‐glycerophosphate, 50 μg/mL^−1^ ascorbic acid, and 200nM hydrocortisone‐21‐hemisuccinate (Sigma‐Aldrich, St. Louis, MO, USA). MK‐4 (10μM) (gift from Kappa Biosciences) and 25(OH)D_3_ (0.01μM; EDQM) were added alone or in combination. Cell medium without vitamins (untreated) was used as control. Culture medium was changed every 3 days. Osteospheres (approximately 2 mm in diameter) were harvested after 21 days and divided into two halves with a scalpel. One half was stored in −80°C until the mechanical testing. The other half was fixed, sectioned, and evaluated by confocal microscopy.

### Mechanical testing of osteospheres

**Fig 1:**
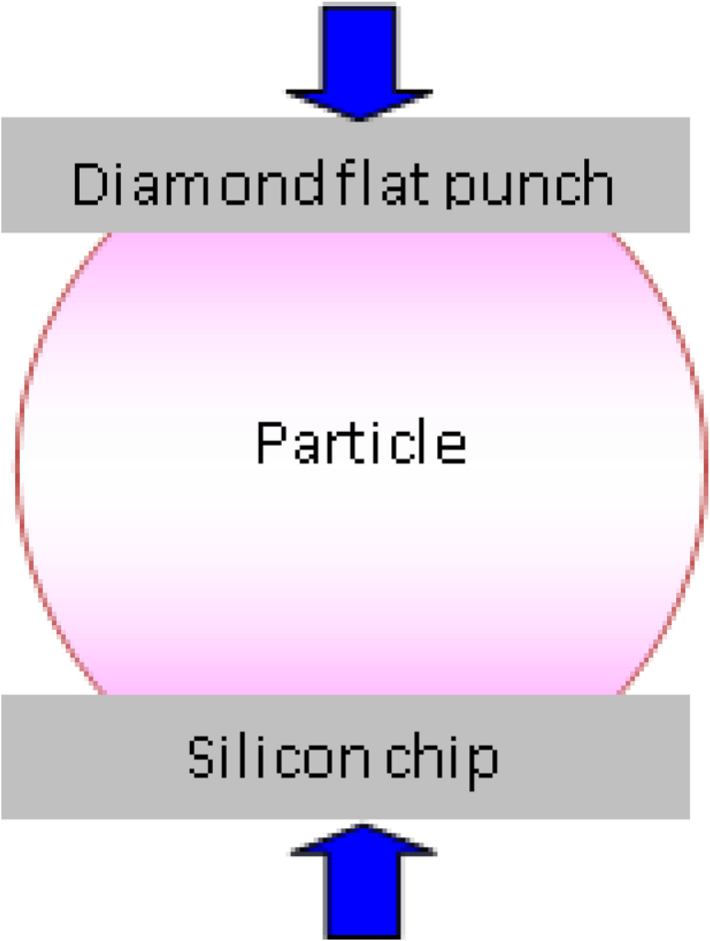
Schematic illustration of the flat‐punch method used for compression test of particle‐like materials.

**Fig 2:**
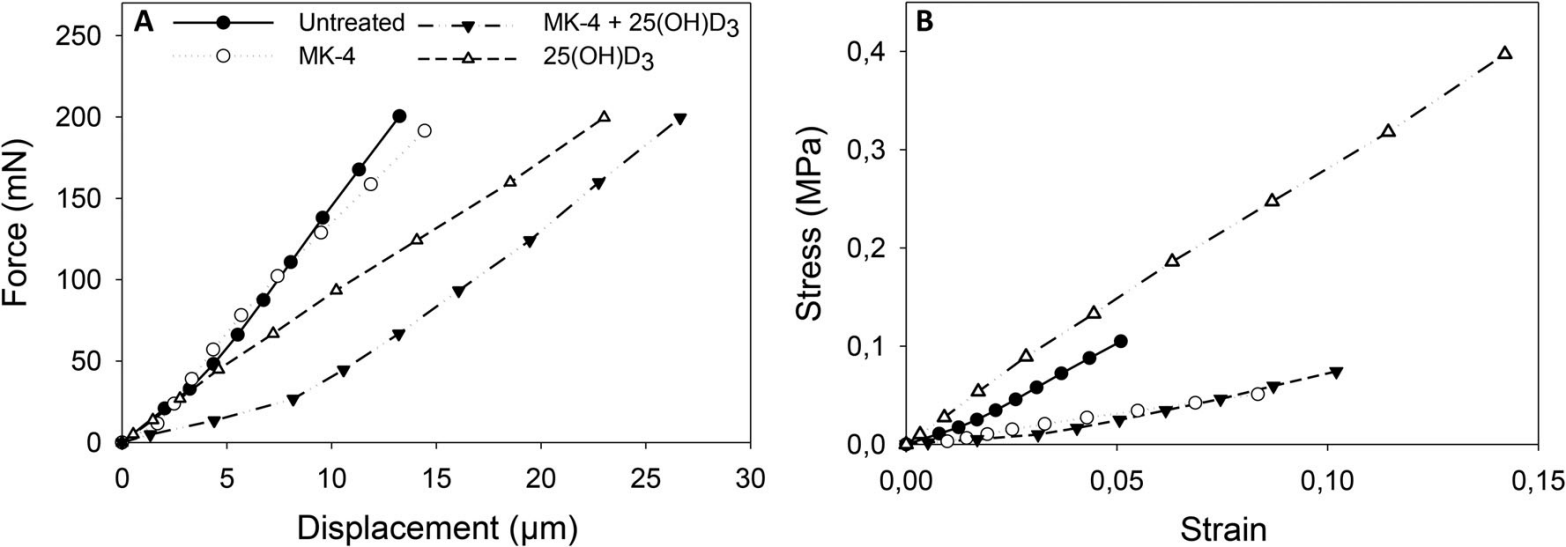
Compression force‐displacement curves and stress–strain relationship from osteospheres of primary human osteoblasts without treatment and treated with 10μM menaquinone‐4 (MK‐4) or 0.01μM 25‐hydroxyvitamin D_3_ [25(OH)D_3_], alone and in combination. (*A*) Shows the global response of the spheres, ie, nanoindentation force versus displacement, the corresponding nominal stress‐strain response is illustrated in (*B*). Notable differences in the stiffness of the vitamin supplemented osteospheres compared with the untreated sample were observed as the tangent stiffness of the 25(OH)D_3_‐treated sample was considerably higher and of the MK‐4 and MK‐4 + 25(OH)D_3_‐treated sample lower than under untreated conditions.

The semispheres were thawed overnight and dried for 24 to 48 hours at room temperature in air. The main global geometry, ie, the surface at the equatorial plane and the height of the samples, was established with a microscope. μCT scanning was not applicable because of the low density of the immature bone tissue. Based on the size of a pixel in the microscope image, the size of the surface was transformed into real size. Assuming an elliptical cross section, a section area was determined and used to calculate the equivalent circular cross section with an equivalent radius. The average cross‐section radius and the height of the samples were applied in finding stress and strain measures from the measured global force and displacement in the mechanical testing of the semiosteospheres. The mechanical response of the osteospheres at room temperature was characterized by nanoindentation using a Hysitron TI950 TriboIndenter (Hysitron, Minneapolis, MN, USA). Because of the irregular geometry of the samples, conventional nanoindentation was not applicable. Instead, a so‐called flat‐punch method for a compression test of the particle‐like materials was used.^(^ ^41^ ^)^ The semispheres were placed on a silicon chip and compressed with a diamond flat punch with a diameter of 1.08 mm, comparable with sample size, as previously illustrated in Haugen and colleagues.^(^ ^6^ ^)^ A sketch of the compression test set‐up is given in Fig. 1. The predefined loading function consisted of one cycle with a small load sequence of maximum 50 mN with a 2‐s hold time at load peak. Then, a 10‐cycle sequence leading up to a 50‐mN maximum load, and finally a 10‐cycle sequence of increasing load up to 200 mN were applied. The cyclic load–displacement response was done stepwise with the load protocol increasing in 10 steps to 200 mN with partial unloadings, as a viscous effect evolves when the peak load is held constant. A nominal measure of tangential stiffness can be estimated by connecting the 10 points corresponding to each load increase. This leads to the response curves, as shown in Fig. 2*A*. To remove some of the geometrical influences of the semispheres on the response, the curves in Fig. 2*A* are mapped into nominal stress and strain. The global load was divided by the equivalent semicircular equatorial cross‐section area to get a stress measure (ie, stress = punch force/π*r* ^2^, where *r* is the radius of the semicircular equatorial cross section). The resultant global displacement was divided by the height of the sample to obtain a strain measure (ie, strain = global displacement/height of the semisphere).

### Microscopy analysis of osteospheres

Osteospheres were washed in sterile PBS, fixed with 4% paraformaldehyde, embedded in OCT frozen sectioning medium (VWR International BVBA, Leuven, Belgium), and sectioned at a thickness of 10 μm using a CryoStar NX70 cryostat (Thermo Fisher Scientific, Waltham, MA, USA). For immunofluorescence characterization, sections were stained with a modified version of Goldner's trichrome method.^(^ ^42^ ^)^ Weigert's hematoxylin solution, chromotrop 2R, fuchsine acid, orange G, tungstophosphoric acid, and fast green powder, as well as Entellan mounting medium were purchased from Merck KGaA (Merck, Darmstadt, Germany). In brief, sections were incubated in Bouin's solution (Sigma‐Aldrich) for 1 hour at 50°C, washed in tap water, stained with Weigert's hematoxylin for 5 min, and washed again. After incubation with chromotrope 2R/fuchsine acid for 15 min, sections were washed in 1% acetic acid, stained with orange G for 7 min, washed in 1% acetic acid, stained with fast green for 10 min, and washed in 1% acetic acid again. After dehydration, the sections were mounted with entellan and imaged with a Leica DM RBE microscope (Leica, Wetzlar, Germany) with a digital camera. Prior to confocal microscopy, the sections were immunostained with primary antibodies against periostin and collagen type I (COL‐1). Antigen retrieval was performed in 10mM citrate buffer, pH 6.0, with 0.05% Tween 20 at 60°C overnight. Sections were cooled to room temperature, washed with PBS, permeabilized with 0.1% triton X‐100 for 10 min, washed with PBS, and blocked in 10% normal goat serum (NGS; Abcam, Cambridge, UK) for 1 hour at room temperature. Sections were then incubated with rabbit antiperiostin (ab14041; Abcam) and mouse COL‐1 (ab90395; Abcam) antibody at a 1:300 dilution in 2% NGS, overnight at 4°C, and washed three times with PBS. Alexa Fluor 488 goat anti‐rabbit (Thermo Fisher Scientific) and Alexa Fluor 568 goat anti‐mouse (Thermo Fisher Scientific) secondary antibodies were used at a 1:500 dilution in 4% NGS for 1 hour at room temperature, sections were washed three times with PBS, counterstained with Hoechst 33342 (1 μg/mL; Sigma‐Aldrich) for 30 min and mounted. Sections were imaged with Leica SP8 confocal microscope (Leica Microsystems CMS GmbH, Mannheim, Germany) using 405‐, 488‐, and 552‐nm excitation, and 420‐ to 480‐nm, 500‐ to 550‐nm, and 580‐ to 630‐nm emission filters for Hoechst 33342, Alexa Fluor 488, and Alexa Fluor 568, respectively. Confocal images were processed with ImageJ software (NIH, Bethesda, MD, USA; https://imagej.nih.gov/ij/). For each image, random ROIs (*n* = 5) on each section were selected and quantified for their mean intensity. Five ROIs were also selected in the image areas containing no section (background), quantified for their mean intensity, averaged, and subtracted from the section mean intensities. 5‐bromo‐4‐chloro‐3‐indolyl phosphate/nitro blue tetrazolium (Sigma‐Aldrich) was applied for detection of alkaline phosphatase (ALP) in frozen sections of osteospheres as previously described by Brauer and colleagues.^(^ ^43^ ^)^

### Alkaline phosphatase activity assay

ALP activity in the cell culture media of the 3D osteospheres after 1, 3, 7, and 14 days of culture was determined by measuring the hydrolysis of p‐nitrophenyl phosphate (pNPP) (Sigma‐Aldrich) into the yellow end‐product p‐nitrophenol, which absorbs at 405 nm. Prior to analysis, aliquots of the cell culture media were concentrated fivefold using MicrosepTM centrifugal tubes with 3 KDa cut‐off from Pall Life Science (Ann Arbor, MI, USA). There was 25 μL of each concentrated sample incubated with 100‐μL pNPP for 30 min in the dark at room temperature; then, the reaction was stopped by adding 50 μL of 3M NaOH. The absorbance was measured at 405 nm in a plate reader (ELX800; BioTek, Winooski, VT, USA) and the ALP activity was quantified using a standard curve based on calf intestinal ALP (Promega, Madison, WI, USA).

### Quantification of proteins secreted in the cell culture medium

Multianalyte profiling of protein levels in the culture media of the 2D cultures and of the osteospheres was performed on the Luminex 200 system employing xMAP technology (Luminex Corp., Austin, TX, USA). Acquired fluorescence data were analyzed by the xPONENT 3.1 software (Luminex). Prior to analysis, aliquots of the cell culture media from the 2D experiment were concentrated 10‐fold using MicrosepTM centrifugal tubes (Pall Life Science) with 3 KDa cut‐off . Analyses were performed using the Milliplex Human Bone Panel kit (EMD Millipore, Billerica, MA, USA). For the 2D cultures, the effect of MK‐4 and 25(OH)D_3_, alone and in combination, on the secretion of cytokines and proteins (IL‐1b, IL‐6, osteoprotegerin [OPG], OC, leptin, osteopontin [OPN], PTH, TNF‐α, adrenocorticotropic hormone, adiponectin, and insulin) to the culture medium after 1, 7, 14, and 20 days were measured. The secretion of OC, OPG, OPN, dickkopf‐related protein 1, FGF23, IL‐6, and sclerostin to the culture medium of the osteospheres was assessed after 1, 3, 7, and 14 days of vitamin treatment. Furthermore, in the 3D experiment, the level of angiogenic markers was determined using the Milliplex Human Angiogenesis / Growth Factor Panel kit (granulocyte‐colony stimulating factor, leptin, VEGF‐A, VEGF‐C, and VEGF‐D). All analyses were performed according to the manufacturer's protocols.

### RNA isolation and RT‐PCR analysis

Total mRNA from 3D osteospheres and 2D cultures was isolated using the Dynabeads mRNA DIRECT kit (Thermo Fisher Scientific ) with some modifications to the manufacturer's protocol. Briefly, the cells were lysed in lysis/binding buffer (100mM Tris–HCl, pH 7.5, 500mM LiCl, 10mM EDTA, pH 8.0, 1% lithium dodecyl sulfate, 5mM dithiothreitol), the lysate was sonicated (UP50H; Hielscher Ultrasonics GmbH, Teltow, Germany) for 10 s and centrifuged for 5 min at 4°C; then the supernatant was collected. mRNA was isolated using magnetic beads [oligo (dT)_25_] as described by the manufacturer. Beads containing mRNA were suspended in 10mM Tris–HCl, pH 7.5, and stored at −80°C until use. Two‐ step RT‐PCR was performed using technical triplicates of total mRNA for the first cDNA Strand Synthesis kit 1612 according to the manufacturer's protocol (Thermo Fisher Scientific). The second step, real‐time PCR was carried out in a Bio‐Rad CFX 384 (Bio‐Rad Laboratories, Hercules, CA, USA), using SYBR green‐based assay iQ SYBR supermix (Bio‐Rad Laboratories). RT‐PCR data were analyzed using the 2^− ΔΔCt ^method 2[−Delta Delta C(T)].^(^ ^44^ ^)^ Each treatment was compared with the respective control and normalized against β‐actin. The primer sequences are listed in Table 1.

**Table 1:** Primer Sequences Used for Real‐Time RT‐PCR Analysis

Protein	Gene	Primer sequence (5′ ‐ 3′)
β‐Actin	h‐ACTB h‐ACTB	f CTGGAACGGTGAAGGTGACA r AAGGGACTTCCTGTAACAA
β2‐Microglobulin	h‐*B2M* h‐*B2M*	f AGCAAGGACTGGTCTTTCTATCTC r CATGTCTCGATCCCACTTAACTATC
Collagen type I alpha 1	h‐*COL1A1* h‐*COL1A1*	f CCAAATCCGATGTTTCTGCT r CATCTCCCCTTCGTTTTTGA
Alkaline phosphatase	h‐*ALPL* h‐*ALPL*	f AGACTGCGCCTGGTAGTTGT r GACAAGAAGCCCTTCACTGC
Osteocalcin	h‐*BGLAP* h‐*BGLAP*	f GCTTCACCCTCGAAATGGTA r GCAAGTAGCGCCAATCTAGG
Osteopontin	h‐*SPP1* h‐*SPP1*	f TGAGGTGATGTCCTCGTCTG r GCCGAGGTGATAGTGTGGTT
Periostin	h‐*POSTN* h‐*POSTN*	f GCCCTGGTTATATGAGAATGGA r ATGCCCAGGTGCCATAAAC
OPG	h‐*OPG* h‐*OPG*	f GTGTCTTGGTCGCCATTTTT r TGGGAGCAGAAGACATTGAA
RANKL	h‐*RANKL* h‐*RANKL*	f GCGCTAGATGACACCCTCTC r CGGGGTGACCTTATGAGAAA

### Statistical analysis

Statistical analysis was performed using SigmaPlot software version 14.0 (Systat Software, San Jose, CA, USA). Data obtained by Luminex analysis and RT‐PCR (ΔΔCt values) were compared between the groups by *t* test or Mann–Whitney *U* test, depending on their normal distribution. Data are presented as percentage of untreated cells (= 100%) at each time point of observation. Mean intensities from the confocal image analysis (*n* = 5 per sample) were compared between the groups by *t* test. A probability of ≤0.05 was considered significant.

## Results

### 25(OH)D_3_ increases and MK‐4 reduces the stiffness of osteospheres

The nominal stress–strain response, illustrating potential effects of the vitamin treatment on the mechanical properties of the irregularly shaped osteospheres, is shown in Fig. 2*B*. The tangent stiffness was obtained by the line connecting the stress at each cyclic peak on the loading part of the stress–strain curves and found to be 1.93 MPa for the untreated semisphere, 0.61 MPa and 2.83 MPa for MK‐4 and 25(OH)D_3_ treatment alone, respectively, and 0.63 MPa for MK‐4 in combination with 25(OH)D_3_.

### MK‐4 alters the expression of periostin and COL‐1 in 3D osteospheres and enhances *POSTN* and *COL1A1* expression in 2D cultures

**Fig 3:**
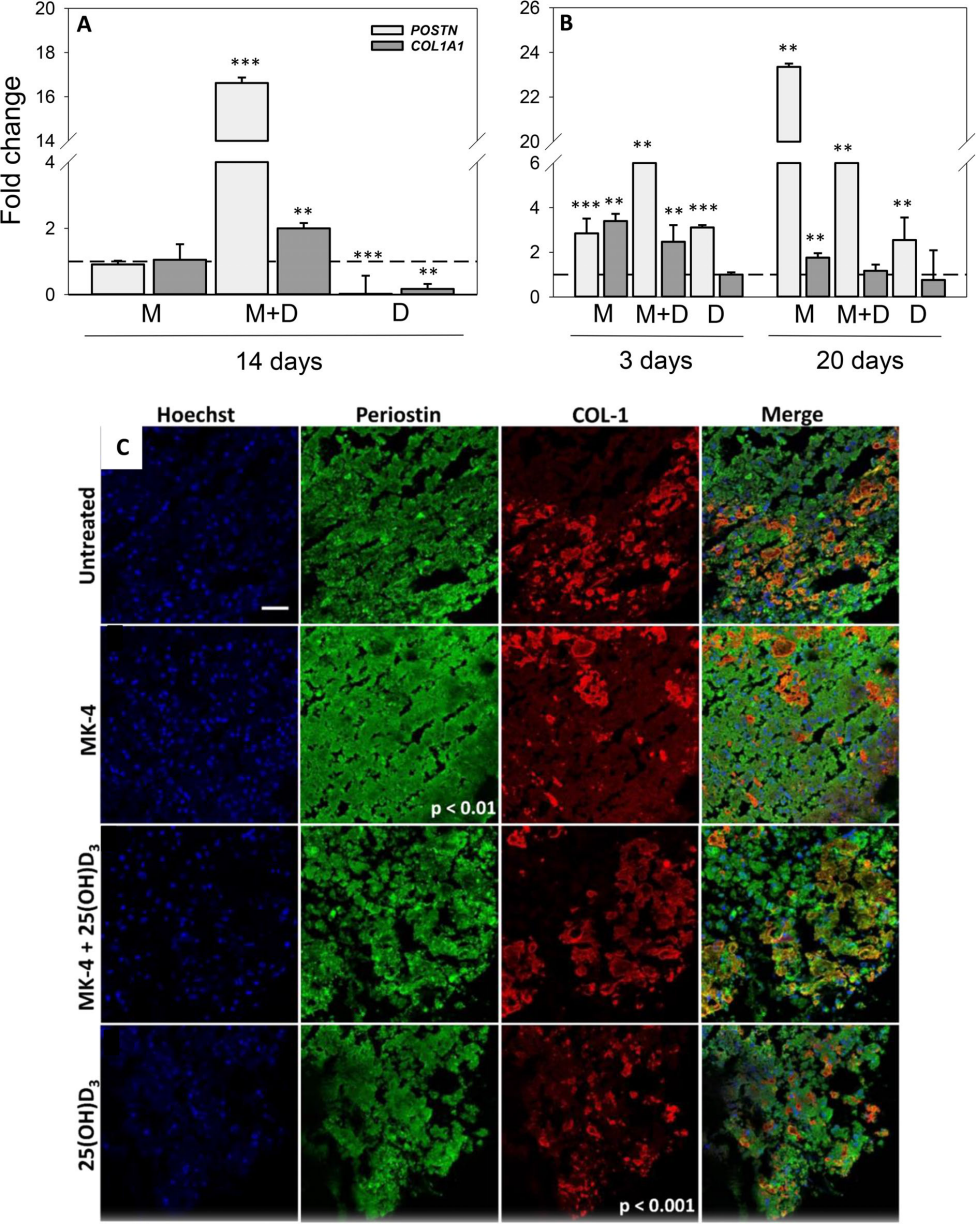
Effect of menaquinone‐4 (MK‐4) and 25‐hydroxyvitamin D_3_ [25(OH)D_3_], alone and combined, on periostin and collagen type I (COL‐1) expression in 3D and 2D cultures of primary human osteoblasts (hOBs): Relative mRNA expression levels for *POSTN* and *COL1A1* in 3D osteospheres (*A*) and 2D cultures of primary hOBs (*B*) cultured with 10μM MK‐4 (M) or 0.01μM 25(OH)D_3_ (*D*), alone and in combination (MD) at different days after vitamin addition. Relative mRNA expression levels were normalized to reference gene *ACTB* (2D cultures) and *ACTB* and *B2M* (3D cultures) and presented as fold‐change relative to unexposed controls. Values represent the mean ± SD. (*C*) Immunofluorescence characterization of cell nuclei (blue), periostin (green), and COL‐1 (red) in selected areas of frozen sections of 21‐day‐old mineralized osteospheres without treatment and treated with 10 μM MK‐4, 10μM MK‐4 and 0.01 μM 25(OH)D_3_, and 0.01μM 25(OH)D_3_ (scale bar = 50 μm). Mean intensities were quantified in five random regions of interest in each whole section. Significant differences were analyzed with SigmaPlot *t* test. Significant different from control at *p* < 0.05, *p* < 0.01, and *p* < 0.001.

Exposure of 3D osteospheres to 25(OH)D_3_ reduced the mRNA expression of *POSTN* 50‐fold (*p* < 0.001) and *COL1A1* more than fivefold (*p* < 0.01) relative to control on day 14. In contrast, the combination of MK‐4 and 25(OH)D_3_ enhanced *POSTN* expression levels more than 16‐fold (*p* < 0.001) and *COL1A1* levels twofold (*p* < 0.01; Fig. 3*A*).

In the 2D cultures, the relative *POSTN* expression on day 3 was elevated 13‐fold by combined vitamin conditions (*p* < 0.01), threefold by 25(OH)D_3_ (*p* < 0.001), and more than twofold by MK‐4 (*p* < 0.001). On day 20, *POSTN* gene expression was enhanced most by exposure to MK‐4 (23‐fold; *p* < 0.01), followed by combined vitamin supplementation (sixfold; *p* < 0.01) and 25(OH)D_3_ (threefold; *p* < 0.01; Fig. 3*B*). Moreover, exposure to MK‐4 increased the relative mRNA expression of *COL1A1* in the 2D cultures threefold (*p* < 0.01) on day 3 and twofold (*p* < 0.01) on day 20. In combination with 25(OH)D_3_, a more than twofold (*p* < 0.01) rise occurred on day 3.

Primary hOBs in frozen sections of 21‐day‐old mineralized 3D osteospheres expressed periostin and produced COL‐1. Interestingly, in the untreated osteospheres, COL‐1 was expressed as a stripe‐like area in the outer regions of the semiconstructs. Osteospheres treated with MK‐4 showed a significant stronger expression of periostin than the control (*p* < 0.01). Additionally, in these osteospheres, COL‐1 was expressed in small amounts over the whole area of the semispheres. Combined administration of MK‐4 and 25(OH)D_3_ did not induce significant changes in COL‐1 and periostin expression or the COL‐1 expression pattern compared with the control. COL‐1 in osteospheres treated with 25(OH)D_3_ alone was expressed at a reduced level (*p* < 0.001), and also all over the area of the semiconstructs compared with the control (Fig. 3*C*).

### 25(OH)D_3_ increases the secretion of ALP from 3D osteospheres and enhances *ALPL* expression in 2D cultures

**Fig 4:**
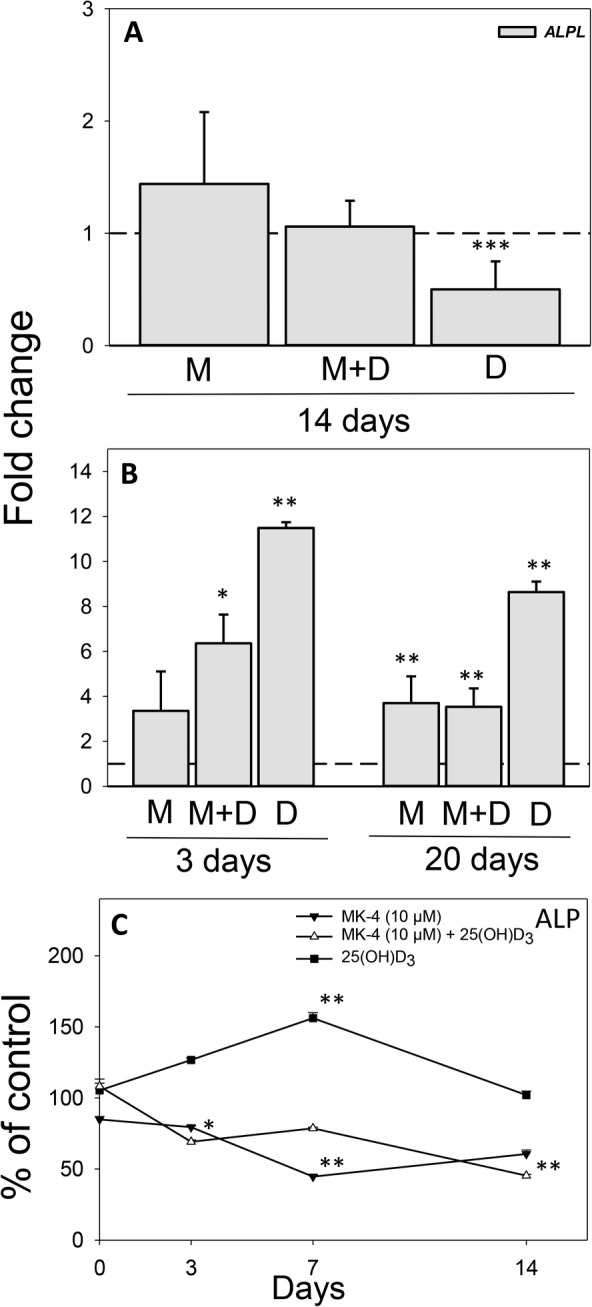
Effect of menaquinone‐4 (MK‐4) and 25‐hydroxyvitamin D_3_ [25(OH)D_3_], alone and combined, on alkaline phosphatase (ALP) in 3D and 2D cultures of primary human osteoblasts (hOBs): Relative mRNA expression levels for *ALPL* in 3D osteospheres (*A*) and 2D cultures of primary hOBs (*B*) cultured with 10 μM MK‐4 (M) or 0.01 μM 25(OH)D_3_ (*D*), alone and in combination (MD) at different days after vitamin addition. Relative mRNA expression levels were normalized to reference gene *ACTB* (2D cultures) and *ACTB* and *B2M* (3D cultures) and presented as fold‐change relative to unexposed controls. (*C*) Secretion of ALP to the culture medium from 3D osteospheres cultured with 10 μM MK‐4 or 0.01μM 25(OH)D_3_, alone and in combination. Spheres were grown for 7 days under untreated conditions, on culture day 8 (= day 0 of comparison to control) vitamins were added to the culture medium. Values represent the mean ± SD. Significant different from control at *p < 0.05, **p < 0.01, and ***p < 0.001.

Exposure of 3D osteospheres to 25(OH)D_3_ reduced the mRNA expression of *ALPL* twofold (*p* < 0.001) relative to control on day 14, whereas no significant differences were observed after exposure to MK‐4 or the vitamins in combination (Fig. 4*A*). Conversely, incubation of 2D cultures with 25(OH)D_3_ increased *ALPL* expression more than 11‐fold (*p* < 0.01) on day 3 and eightfold (*p* < 0.01) on day 20. In addition, relative *ALPL* expression was enhanced sixfold (*p* < 0.05) by MK‐4 and 25(OH)D_3_ together on day 3, and more than threefold by both MK‐4 alone and the combination on day 20 (*p* < 0.01 for both; Fig. 4*B*).

The levels of membrane‐bound ALP in frozen sections of 21‐day‐old mineralized 3D osteospheres were not affected by any of the vitamins compared with control (data not shown). However, the secretion of ALP to the culture medium from 3D osteospheres was decreased to 70 ± 1.3% (*p* < 0.05) of control on day 3 and 45 ± 0.8% (*p* < 0.01) on day 14 by combined supplementation with MK‐4 and 25(OH)D_3_. Similarly, ALP secretion was reduced to 45 ± 1% (*p* < 0.01) of control by MK‐4 on day 7. In contrast, a rise in ALP secretion to 156 ± 4% (*p* < 0.01) of control was observed on day 7 after exposure to 25(OH)D_3_ (Fig. 4*C*).

### 25(OH)D_3_ enhances the deposition of mineral in osteospheres

**Fig 5:**
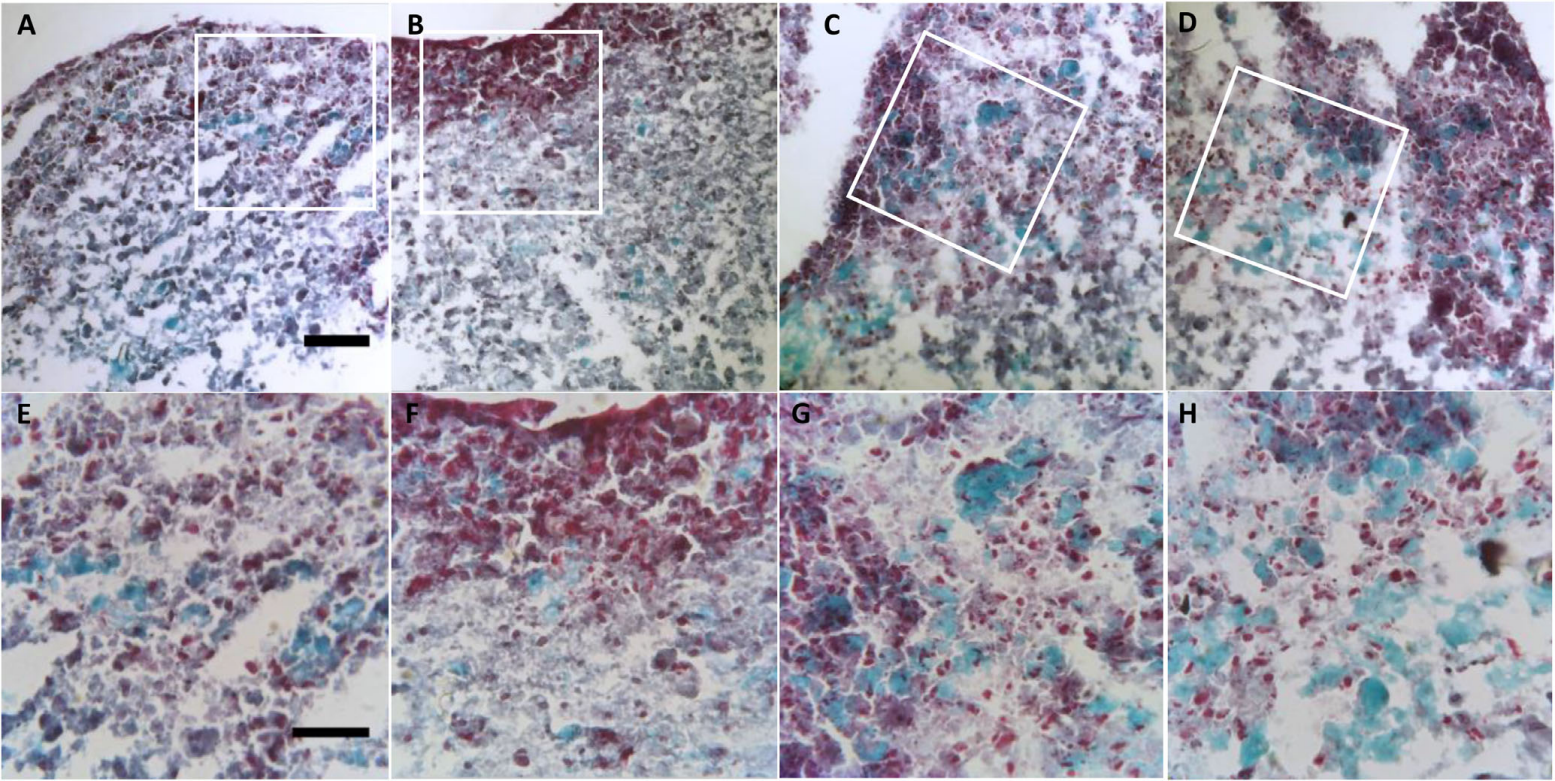
Histochemical characterization of cell nuclei (black), osteoid (red), and mineralized bone (green) stained with Goldner trichrome method in frozen sections of 21‐day‐old mineralized osteospheres. (*A* to *D*) Show a 10‐μm frozen section of a semiosteosphere without treatment (*A*), treated with 10μM MK‐4 (*B*), 10μM MK‐4 + 0.01μM 25‐hydroxyvitamin D_3_ [25(OH)D_3_] (*C*), or 0.01μM 25(OH)D_3_ (*D*) (scale bar = 100 μm). (*E* to *H*) Are high‐magnification images (scale bar = 50 μm) of a representative area of semiosteospheres without treatment (*E*), treated with 10μM MK‐4 (*F*), 10μM MK‐4 + 0.01μM 25(OH)D_3_ (*G*), or 0.01 μM 25(OH)D_3_ (*H*).

Frozen sections of untreated 21‐day‐old mineralized bone spheroids showed large osteoid formation in the outer region of the semiconstructs, whereas little deposition of mineral was detected within the spheres (Fig. 5*A*,*E*). MK‐4 supplementation did not affect mineralization, but the osteoid appeared to be much more condensed compared with the control (Fig. 5*B*,*F*). Osteospheres treated with a combination of MK‐4 and 25(OH)D_3_ showed extended osteoid formation into the central region of the constructs and increased mineral deposition over the whole area compared with the control (Fig. 5*C*,*G*). In osteospheres treated with 25(OH)D_3_ alone, increased mineralization organized as a stripe‐like area over the semiconstruct was observed (Fig. 5*D*,*H*).

### 25(OH)D_3_ alone, or in combination with MK‐4 alters the secretion of OC and IL‐6 of primary hOBs

**Fig 6:**
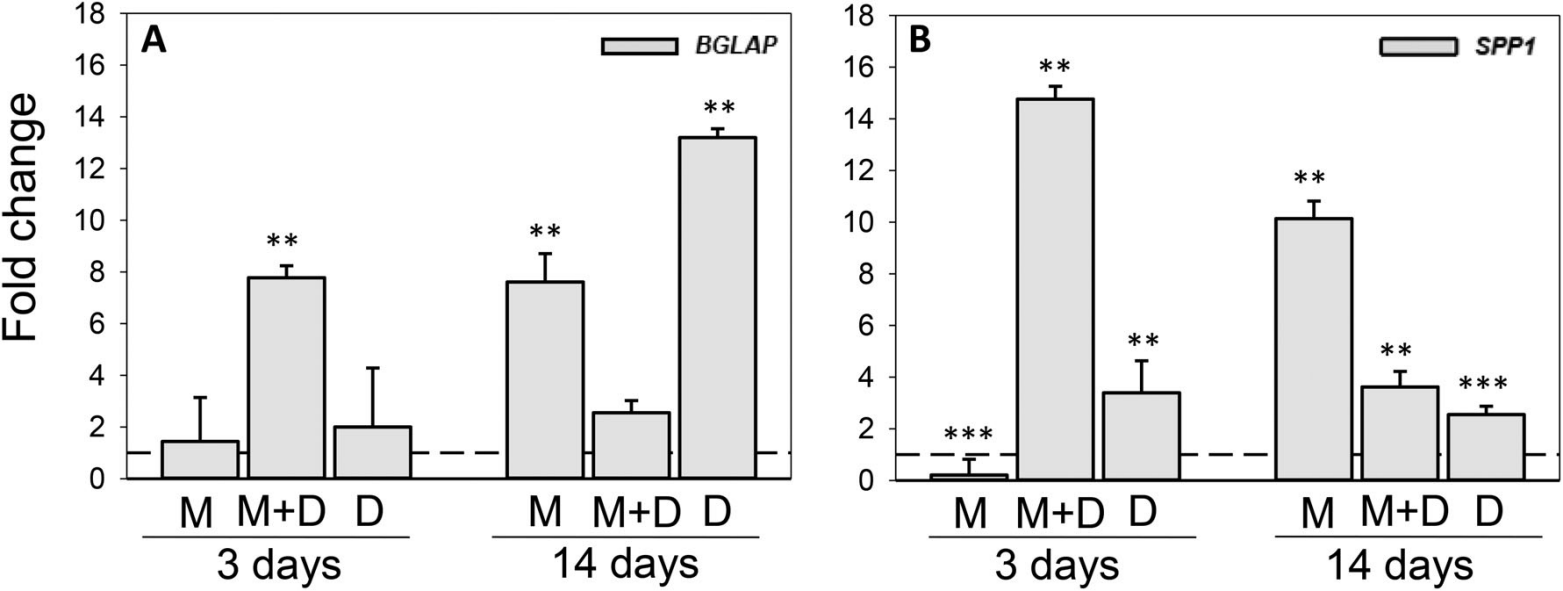
Effect of menaquinone‐4 (MK‐4) and 25‐hydroxyvitamin D_3_ [25(OH)D_3_], alone and combined, on osteocalcin (*BGLAP*) and osteopontin (*SPP1*) gene expression in 2D cultures of primary human osteoblasts: Relative mRNA expression levels for *BGLAP* (*A*) and *SPP1* (*B*) in 2D cultures exposed to 10μM MK‐4 (M) or 0.01 μM 25(OH)D_3_ (*D*), alone and in combination (MD) at days 3 and 20 after vitamin addition. Relative mRNA expression levels were normalized to reference gene *ACTB* and presented as fold‐change relative to unexposed controls. Values represent the mean ± SD. Significant different from control at *p < 0.05, **p < 0.01, and ***p < 0.001.

In the 2D cultures, combined supplementation with MK‐4 (10μM) and 25(OH)D_3_ significantly increased the relative mRNA expression of *BGLAP* more than sevenfold (*p* < 0.01) on day 3. Additionally, *BGLAP* expression levels were raised 13‐fold (*p* < 0.01) by exposure to 25(OH)D_3_ and eightfold (*p* < 0.01) by MK‐4 on day 20 (Fig. 6*A*). Relative *SPP1* expression on day 3 was elevated more than 14‐fold by combined vitamin conditions (*p* < 0.01) and threefold by 25(OH)D_3_ (*p* < 0.01), whereas MK‐4 (10μM) reduced the expression fivefold (*p* < 0.001). On day 20, *SPP1* gene expression was enhanced most by exposure to MK‐4 (10‐fold; *p* < 0.01), followed by combined vitamin supplementation (fourfold; *p* < 0.01) and 25(OH)D_3_ (threefold, *p* < 0.001; Fig. 6*B*).

**Fig 7:**
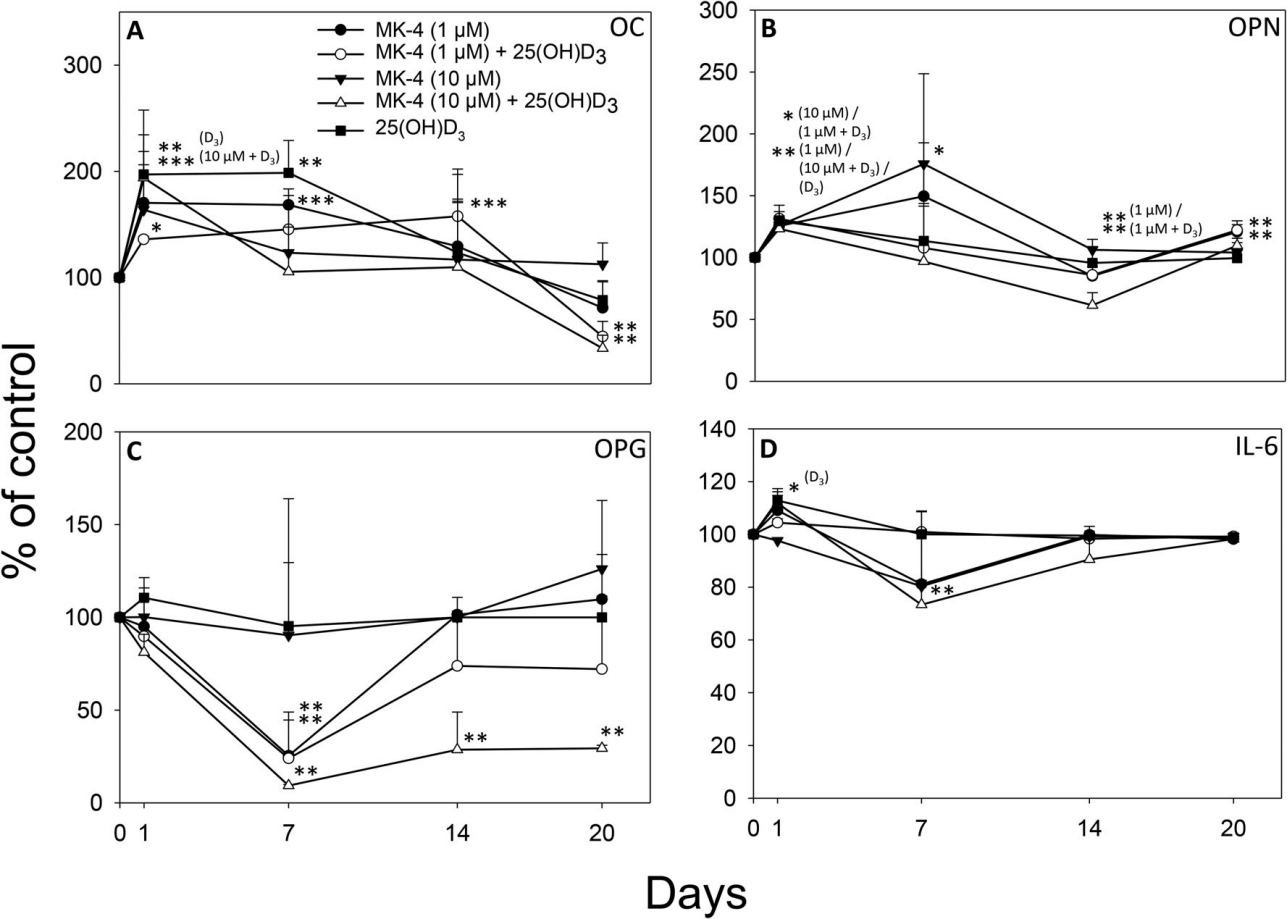
Secretion of osteocalcin (OC) (*A*), osteopontin (OPN) (*B*), osteoprotegrin (OPG) (*C*), and interleukin‐6 (IL‐6) (*D*) to the culture medium from primary human osteoblasts treated with 1μM menaquinone‐4 (MK‐4), 10μM MK‐4 or 0.01μM 25‐hydroxyvitamin D_3_ [25(OH)D_3_], alone and in combination, is shown in % of control at 1, 7, 14, and 20 days. Values represent the mean ± SD. Significant different from control at *p < 0.05, **p < 0.01, and ***p < 0.001.

Protein levels of IL‐1b, leptin, TNF‐α, and adiponectin in the cell culture media of the 2D cultures were below the detection limit for the standard curves and are consequently not presented. Both 25(OH)D_3_ alone and in combination with MK‐4 (1μM and 10μM) resulted in an acute increased secretion of OC (day 1: 197 ± 61%, *p* < 0.01; 136 ± 3%, *p* < 0.05; and 194 ± 12%, *p* < 0.001, respectively). The OC release was elevated by both 25(OH)D_3_ and MK‐4 (1μM) alone on day 7 (199 ± 31%, *p* < 0.01% and 168 ± 1.4%, *p* < 0.001, respectively) and by the combined treatment of MK‐4 (1μM) and 25(OH)D_3_ on day 14 (158 ± 16%, *p* < 0.001) compared with control. On day 20, OC levels were reduced to around 40% of control by the combined administration of MK‐4 (both 1μM and 10μM) and 25(OH)D_3_ (*p* < 0.01 and *p* < 0.01, respectively; Fig. 7*A*). The amount of OPN in the culture medium was increased by 30% over control by all treatments after one day of incubation. After 7 days, OPN levels were only significantly higher for MK‐4 (10μM) treatment (176 ± 17%, *p* < 0.05) and the release was reduced by MK‐4 (1μM) alone and in combination with 25(OH)D_3_ on day 14 (85 ± 12% and 86 ± 7%, respectively: *p* < 0.01 for both; Fig. 7*B*). OPG levels were fourfold reduced by MK‐4 (μM) alone (*p* < 0.01) and in combination with 25(OH)D_3_ (*p* < 0.01) 7 days after treatment. Combined supplementation of MK‐4 (10μM) and 25(OH)D_3_ decreased the secretion of OPG to 9 ± 0.6% at day 7 (*p* < 0.01) and maintained the reduced secretion of OPG to 29 ± 1.7% of control at day 20 (*p* < 0.01; Fig. 7*C*). The release of IL‐6 was significantly enhanced by 25(OH)D_3_ on day 1 (113 ± 4%, *p* < 0.05) and reduced in combination with MK‐4 (10μM) on day 7 (73 ± 9%, *p* < 0.01; Fig. 7*D*).

### MK‐4 alone, or in combination with 25(OH)D_3_, alters the expression of *BGLAP*, *SPP1*, *RANKL*, and *OPG* in 3D osteospheres as compared with 25(OH)D_3_ alone

**Fig 8:**
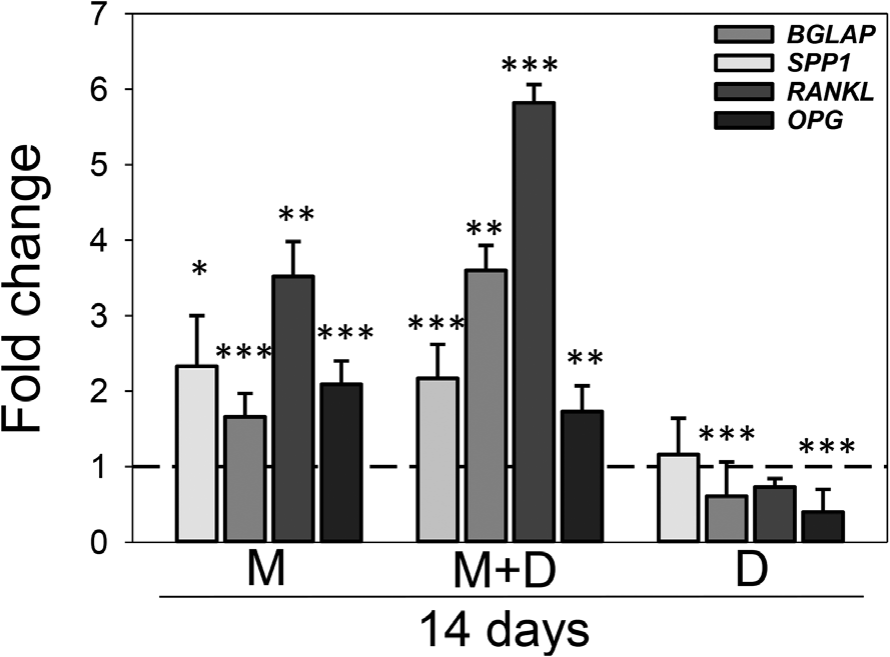
Effect of menaquinone‐4 (MK‐4) and 25‐hydroxyvitamin D_3_ [25(OH)D_3_], alone and combined, on osteocalcin (*BGLAP*), osteopontin (*SPP1*), osteoprotegrin (*OPG*), and receptor activator of nuclear factor‐κB ligand (RANKL) gene expression in 3D cultures of primary human osteoblasts: Relative mRNA expression levels for, *BGLAP*, *SPP1*, *OPG*, and *RANKL* in 3D osteospheres cultured with 10μM MK‐4 (M) or 0.01μM 25(OH)D_3_ (D), alone and in combination (MD) at day 14 after vitamin addition. Relative mRNA expression levels were normalized to reference genes *ACTB* and *B2M* and presented as fold‐change relative to unexposed controls. Values represent the mean ± SD. Significant different from control at *p < 0.05, **p < 0.01, and ***p < 0.001.

Exposure of 3D osteospheres to 25(OH)D_3_ did not significantly change *BGLAP* and *RANKL* gene expression, but reduced *SPP1* expression 1.6‐fold (*p* < 0.001) and *OPG* expression 2.5‐fold (*p* < 0.001) on day 14. Conversely, treatment with MK‐4 increased the relative mRNA expression of *BGLAP* more than twofold (*p* < 0.05), of *SPP1* 1.6‐fold (*p* < 0.001), of *RANKL* more than threefold (*p* < 0.01) and of *OPG* more than twofold (*p* < 0.001). Similarly, *BGLAP* expression levels were raised twofold (*p* < 0.001), *SPP1* levels more than threefold (*p* < 0.01), *RANKL* levels more than fivefold (*p* < 0.001), and *OPG* levels 1.7‐fold (*p* < 0.01) by combined vitamin conditions (Fig. 8). In addition, the *RANKL/OPG* ratio was significantly downregulated in 3D osteospheres treated with 25(OH)D_3_ (1.59, *p* < 0.001), as well as both vitamins in combination (1.80, *p* < 0.01) compared with control (3.05). MK‐4 alone did not significantly affect the *RANKL/OPG* ratio (3.36, *p* > 0.05) after 14 days.

### MK‐4 alone or in combination with 25(OH)D_3_ alters the secretion of OPG, VEGF‐C, IL‐6, and G‐CSF from 3D osteospheres as compared with 25(OH)D_3_ alone

**Fig 9:**
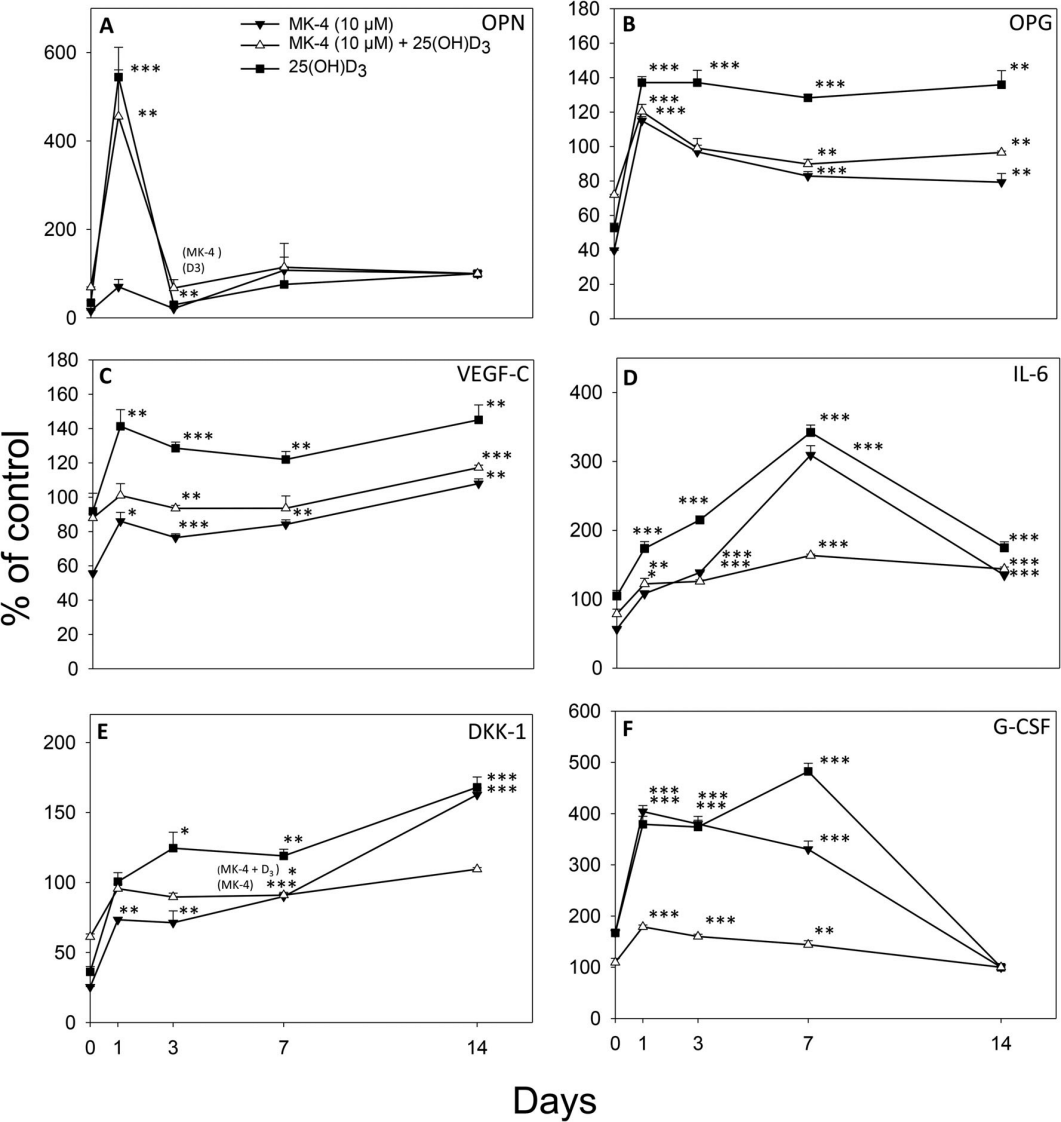
Secretion of osteopontin (OPN) (*A*), osteoprotegrin (OPG) (*B*), vascular endothelial growth factor C (VEGF‐C) (*C*), interleukin‐6 (IL‐6) (*D*), dickkopf‐related protein 1 (DKK1) (*E*), and granulocyte‐colony stimulating factor (G‐CSF) (*F*) to the culture medium from osteospheres of human primary osteoblasts treated with 10μM menaquinone‐4 (MK‐4) or 0.01μM 25‐hydroxyvitamin D_3_ [25(OH)D_3_], alone and in combination, is shown in % of control at 1, 3, 7, and 14 days. Spheres were grown for 7 days under untreated conditions, on culture day 8 (= day 0 of comparison to control) vitamins were added to the culture medium. Values represent the mean ± SD. Significant different from control at *p < 0.05, **p < 0.01, and ***p < 0.001.

Protein levels of OC, sclerostin, and FGF23 in the cell culture media from osteospheres were below the detection limit for the standard curves and are consequently not presented. Both 25(OH)D_3_ supplementation alone and in combination with MK‐4 (10μM) induced an acute increase in the release of OPN (day 1: 545 ± 67%, *p* < 0.001 and 455 ± 105%, *p* < 0.01, respectively), whereas MK‐4 alone had no significant effect compared with untreated control cells. On day 3, OPN levels were reduced to around 20% of control by both single treatments with MK‐4 and 25(OH)D_3_ (*p* < 0.01 and *p* < 0.01, respectively), and afterwards stabilized to near control levels (Fig. 9*A*). The secretion of OPG was increased by 25(OH)D_3_ alone to approximately 40% of control during the whole culture period. MK‐4 (10μM) alone and in combination with 25(OH)D_3_ significantly enhanced the OPG release on day 1 (*p* < 0.001) and reduced it on day 14 (*p* < 0.01) by <20% of control (Fig. 9*B*). Single‐vitamin treatments, or the combination, induced only minor changes into the secretion of VEGF‐C. VEGF‐C levels were 145 ± 9% (*p* < 0.01) for 25(OH)D_3_ alone, 117 ± 2% (*p* < 0.01) for combined vitamin conditions, and 108 ± 3% for MK‐4 compared with the control after 14 days (Fig. 9*C*). The administration of 25(OH)D_3_ significantly enhanced the IL‐6 levels at days 1 and 3 after vitamin treatment (174 ± 10%, *p* < 0.001 and 215 ± 6%, *p* < 0.001, respectively) with peak effect after 7 days (342 ± 11%, *p* < 0.001). MK‐4 treatment alone increased the IL‐6 release threefold (*p* < 0.001) and in combination with 25(OH)D_3_ more than 1.5‐fold (*p* < 0.001) by day 7 (Fig. 9*D*). Significantly higher amounts of DKK‐1 in the culture medium were detected for 25(OH)D_3_ alone at 3 days (126 ± 11%, *p* < 0.05) and 7 days (119 ± 5%, *p* < 0.01) after incubation, and for both single treatments with 25(OH)D_3_ and MK‐4 by day 14 (168 ± 5%, *p* < 0.001 and 163 ± 7%, *p* < 0.001, respectively; Fig. 9*E*). G‐CSF levels were more than threefold enhanced at days 1 (*p* < 0.001) and 3 (*p* < 0.001) by treatment with 25(OH)D_3_ alone. Combined vitamin conditions enhanced the secretion to 179 ± 4% (day 1, *p* < 0.001) and 160 ± 4% (day 3, *p* < 0.001) compared with control. After 7 days, 25(OH)D_3_ administration had peak effect (482 ± 16%, *p* < 0.001), whereas G‐CSF release from MK‐4‐and combined vitamin‐treated cells did not increase further compared with days 1 and 3 (331 ± 16%, *p* < 0.001 and 144 ± 8%, *p* < 0.01, respectively; Fig. 9*F*).

## Discussion

We demonstrate the differential effects of vitamin D and K2 on the mechanical properties of human 3D bone spheroids in vitro ÷ exposure to 25(OH)D_3_‐induced increased stiffness, whereas the synthetic vitamin K2, MK‐4, induced softer or more flexible osteospheres compared with untreated spheroids. Osteospheres treated with a combination of 25(OH)D_3_ and K2 had the same flexibility as those treated with K2 alone. To the best of our knowledge, this is the first study to show that vitamin K2 modulates vitamin D‐induced mechanical properties in a 3D bone model based on hOBs.

Cell‐based in vitro models, previously generated by a rotational coculture approach of hOBs and osteoclasts without any exogenous scaffolding material,^(^ ^1^, ^2^, ^6^ ^)^ enable us to study the bone microenvironment. In contrast to these two cell systems, we produced 3D mineralized tissue constructs from single cultures of primary hOBs. Osteoblasts in our 3D bone spheroids formed mineralized bone matrix similar to Clark and colleagues and Haugen and colleagues,^(^ ^1^, ^6^ ^)^ and secreted the bone matrix protein OPN as reported by Penolazzi and colleagues.^(^ ^2^ ^)^ In addition to 3D spheroids, we applied 2D cultures of primary hOBs to assess the effect of MK‐4 and 25(OH)D_3_ on proteins and cytokines involved in the mechanical and biological function of bone. In 3D cultures, cellular behavior reflects in vivo tissue functionality more accurately than in monolayer cultures. 3D cultures are thus better suited for the evaluation of cellular responses to various compounds or drugs.^(^ ^45^ ^)^

The strength of bone and its ability to resist fracture are dependent on its mass and geometry, but also on the bone material properties,^(^ ^46^ ^)^ which are determined by, among others, the quality, amount, and orientation of collagen fibers^(^ ^47^ ^)^; and degree of mineralization.^(^ ^48^ ^)^ The mineral phase of bone influences the ability to resist deformation and provides stiffness and strength to the bone structure,^47^, ^48^ ^)^ whereas collagen is associated with its flexibility (toughness), giving resistance to impact load.^(^ ^47^, ^49^ ^)^

We observed an increase in ALP secreted to the culture medium from osteospheres exposed to 25(OH)D_3_ and in line with this, enhanced mineral deposition, which may, in addition to the reduced expression of COL‐1, account for their higher bone stiffness. On the other hand, administration of MK‐4 alone reduced the ALP activity in the medium and did not stimulate mineralization, but induced enhanced expression of periostin and altered distribution of COL‐1. This was reflected in reduced bone stiffness and higher flexibility in the osteospheres. In agreement, we found significantly upregulated expression of *POSTN* and *COL1A1* in 2D cultures exposed to MK‐4. However, mRNA expression in the osteospheres was not altered. Periostin is a vitamin K‐dependent protein primarily produced and secreted by osteoblasts and their precursor cells.^(^ ^21^ ^)^ It is an important mediator of the biomechanical properties of collagen‐rich tissues by regulating collagen fibril diameter and cross‐linking.^(^ ^24^ ^)^

In the present study, increased flexibility of the osteospheres was observed after exposure to the two vitamins despite enhanced mineralization. The improvement of flexibility could be attributed to increased synthesis of periostin and COL‐1. Accordingly, *POSTN* and *COL1A1* gene expression levels in these osteospheres were significantly enhanced; however, no evident alterations in the protein levels of periostin and COL‐1 were revealed. Still, it is reasonable that MK‐4 may have facilitated the formation of more collagen with proper physiological function in the osteospheres. Vitamin K2 has been suggested to promote collagen accumulation in osteoblastic cells via the SXR‐signaling pathway.^(^ ^50^ ^)^ Enhanced collagen mRNA expression has also been reported in 2D cultures of osteogenically differentiated human mesenchymal stem cells from amniotic fluid treated with MK‐4; however, protein levels in 3D spheroid cultures were not affected.^(^ ^51^ ^)^ Vitamins D and K2, as well as the combination of the two, have previously been described to enhance mineralization of osteoblasts in vitro.^(^ ^52^ ^)^

In clinical studies, combined administration of vitamins D and K is suggested to improve bone quality and lower the risk of fractures.^(^ ^33^ ^)^ Moreover, a higher gain in BMD has been reported in postmenopausal women with osteoporosis treated with a combination of the vitamins compared with each vitamin alone or calcium.^(^ ^38^, ^39^ ^)^

It is worth noting that the generated osteospheres in our study may comprise osteoblasts in various differentiation stages, similar to the in vivo situation. As previously reported, spheroids with a diameter of 500 μm are made‐up of a heterogenic population of cells, depending upon the location within the layer‐like structure of the sphere.^(^ ^53^, ^54^ ^)^ In the outer rim of a sphere, cells are surrounded by media and have the space to proliferate, whereas cells in the inner area have cell‐to‐cell contact and are dependent on nutrient transport from neighboring cells.^(^ ^54^, ^55^ ^)^ In contrast to the even periostin staining in our study, immunostaining of unexposed osteospheres revealed COL‐1 expressing cells in the outer region of the semispheres. This may indicate that these cells are less differentiated, producing higher amounts of COL‐1.^(^ ^56^ ^)^ The absence of COL‐1 expression in the inner region of our osteospheres suggests that these cells are of later osteoblast or early osteocyte differentiation stage,^(^ ^57^ ^)^ as COL‐1 is downregulated when osteoblasts begin to develop into osteocytes in vitro.^(^ ^56^, ^58^ ^)^ As recently suggested by Kim and Adachi, the cell condensation within spheroids triggers the differentiation of osteoblast‐precursor cells to osteocyte‐like cells.^(^ ^59^ ^)^ The uneven differentiation of osteoblasts in 3D cultures has been previously reported by others.^(^ ^57^, ^60^ ^)^ Alterations in this differentiation pattern within the osteospheres, induced by the vitamins, are reflected in the immunostaining and gene expression analysis.

OC and OPN are major noncollagenous proteins (NCPs) involved in bone matrix organization and deposition, and have been shown to influence bone morphology and mechanical properties.^(^ ^61^ ^)^ Both proteins interact with collagen and mineral.^23^, ^62^ ^)^ It has been recently suggested that their spatial arrangement in the bone matrix enhances bone toughness.^(^ ^63^ ^)^ Among these NCPs, OPN has been proposed to act as a glue that counteracts the separation of the mineralized collagen fibers upon mechanical loading of bone. In this structure, energy may be dissipated through the formation and reformation of intramolecular bonds between OPN and divalent Ca^2+^, which increases the total energy to fracture bone.^(^ ^64^, ^65^ ^)^ Moreover, NCPs influence the mechanical properties of bone through dilatational band formation as suggested by Poundarik and colleagues.^(^ ^63^ ^)^ Dilatational bands are ellipsoidal voids that result from the disassembly of noncollagenous protein complexes, like OC‐OPN complexes, which are integrated in the mineralized matrix of bone when a load is applied. Formation of these microcracks within bone allows for the dissipation of large amounts of energy, which reduces the bone's propensity to fracture.^(^ ^63^ ^)^ The enhanced *BGLAP* and *SPP1* expressions in osteospheres exposed to MK‐4 alone and combined with 25(OH)D_3_, as well as the acute increase in OPN secretion, suggest that the reduced stiffness seen in these osteospheres may be partially mediated by these mechanisms.

The carboxylated form of OC facilitates deposition of calcium into the bone matrix.^(^ ^66^ ^)^ Both vitamins D and K stimulate synthesis of OC and are also cofactors in the carboxylation,^(^ ^37^ ^)^ thereby contributing to mineralization.^(^ ^67^, ^68^, ^69^ ^)^ In line with this, we observed a rise in OC levels in the 2D cell cultures after 1 and 7 days of vitamin D administration, and after 7 days of exposure to vitamin *K*, no further enhancement occurred when combining the two vitamins.

OPN release in 2D cell cultures was promoted by both vitamins after one day of exposure, but only by the higher concentration of MK‐4 (10μM) after 7 days. Vitamin D alone and in combination with MK‐4 also induced a transient increase in OPN in 3D osteospheres.

Based on our findings, it is reasonable that the effects of vitamins D and K are partly mediated by these proteins. It is worth noting that we were not able to detect OC in the culture medium of the osteospheres. This could be attributed to the fact that OC is expressed late in the osteoblast maturation process,^(70^ ^)^ and therefore not detectable in the medium after a culture period of 14 days.

The rate of bone turnover is another determinant of bone quality. Thus, we assessed the impact of the two vitamins on substances regulating bone metabolism. In 2D cultures, MK‐4 administration alone and combined with 25(OH)D_3_ induced a decline in OPG. In contrast, a sustained increase in OPG by exposure to 25(OH)D_3_, as well as a decreased *RANKL/OPG* ratio in 3D spheroids by 25(OH)D_3_ and combined vitamins was seen. These findings may translate to suppression of bone resorption in vivo. In the 3D spheroids, both vitamins induced a rise in IL6. However, data on the effect of IL‐6 on bone metabolism are diverging.^(^ ^71^ ^)^ Moreover, both vitamins induced an increase in DKK1, an inhibitor of bone formation,^(^ ^72^ ^)^ 25(OH)D_3_ at several time points, MK‐4 and the combination of the vitamins only after 14 days. Finally, G‐CSF levels were enhanced by treatment with 25(OH)D_3_ alone, and to a lesser degree by the combined vitamins compared with control. In summary, the two vitamins induced a rise both in factors stimulating and inhibiting bone resorption, as well as factors favoring and inhibiting bone formation. How this translates to in vivo conditions is, however, impeded by the fact that the osteospheres only contained osteoblasts. We observed enhancement of osteoblast differentiation by 25(OH)D_3_ and MK‐4 alone and in combination, as reflected in increased OC levels. Given the interplay between osteoblasts and osteoclasts, the presence of both cells would have given a more complete picture. Still, based on our results, it can be hypothesized that combined administration of K2 and 25(OH)D_3_ could contribute to stronger bone also in vivo. This should be tested in 3D osteospheres containing both osteoblasts and osteoclasts, as well as in rodents and humans.

## Disclosures

MS, EAR, JH, BHS, AML, US, and JER state that they have no conflicts of interest. MEM is a shareholder in Axial Vita AS, which sells vitamin K2. JER is a member of Cost Action 16119 CellFit.

## Author Contributions

**Maria Schroeder:** Formal analysis; investigation; methodology; visualization; writing‐original draft; writing‐review and editing. **Elisabeth Riksen:** Formal analysis; visualization; writing‐original draft. **Jiannying He:** Formal analysis; investigation; visualization; writing‐original draft. **Bjørn Skallerud:** Formal analysis; resources; supervision; writing‐review and editing. **Mona Møller:** Resources; writing‐review and editing. **Aina Lian:** Formal analysis; investigation. **Unni Syversen:** Writing‐original draft; writing‐review and editing. **Janne Reseland:** Conceptualization; funding acquisition; methodology; project administration; supervision; validation; writing‐original draft; writing‐review and editing.

### Peer Review

The peer review history for this article is available at https://publons.com/publon/10.1002/jbm4.10394.
